# Supplementary material for: Collective excitations of a bound-in-the-continuum condensate
Source: Nat Commun. 2023 Jun 12;14:3464. doi: 10.1038/s41467-023-38939-y (PMC10261147; doi:10.1038/s41467-023-38939-y)
Supplement: Supplementary file 1 — Supplementary Information [file 41467_2023_38939_MOESM1_ESM.pdf]

Collective excitations of a bound-in-the-continuum condensate
Supplementary Information

Anna Grudinina,[1] Maria Efthymiou-Tsironi,[2,3] Vincenzo Ardizzone,[2,3] Fabrizio Riminucci,[4] Milena De Giorgi,[3] Dimitris Trypogeorgos,[3] Kirk Baldwin,[5] Loren Pfeiffer,[5] Dario Ballarini,[3] Daniele Sanvitto,[3,*] and Nina Voronova[1,*]

[1]*National Research Nuclear University MEPhI (Moscow Engineering Physics Institute), 115409 Moscow, Russia*
[2]*Dipartimento di Matematica e Fisica "Ennio De Giorgi", Università del Salento, Strada Provinciale Lecce-Monteroni, Campus Ecotekne, Lecce 73100, Italy*
[3]*CNR Nanotec, Institute of Nanotechnology, via Monteroni, 73100, Lecce, Italy*
[4]*Molecular Foundry, Lawrence Berkeley National Laboratory, One Cyclotron Road, Berkeley, California, 94720, USA*
[5]*PRISM, Princeton Institute for the Science and Technology of Materials, Princeton University, Princeton, New Jersey 08540, USA*

In these Supplementary Notes and Supplementary Figures, we provide a detailed derivation of the bare particle dispersions used in the main text when calculating the excitation spectrum, more experimental data on time-resolved and energy-resolved photoluminescence maps, and analysis of the nonradiative losses influence on the system. Additionally, we check the applicability of the Bogoliubov approximation for the parameters used in simulations.

SUPPLEMENTARY NOTES

Exciton-polariton dispersions. For a waveguide with grating shown schematically in Fig. 1a of the main text, the wavevector of the two guided photon modes in the vicinity of the Γ-point reads (see e.g. [1])

$$\boldsymbol{\beta}_\pm = \left(k_x \pm \frac{2\pi}{a}\right)\mathbf{e}_x + k_y\mathbf{e}_y.$$

Corresponding photonic modes dispersions have the form:

$$\hbar\omega_\pm(k_x,k_y) \approx \omega_0 \pm \frac{\hbar c k_x}{n_{\rm g}} + \frac{\hbar c a}{4\pi n_{\rm g}}k_y^2,$$

where a is the period of grating, $n_{\rm g}$ the guided-mode refractive index of the medium (here GaAs), and c the velocity of light in vacuum. The Hamiltonian of the two coupled photonic modes reads:

$$\hat{H} = \begin{pmatrix} \hbar\omega_+ & U \\ U & \hbar\omega_- \end{pmatrix} - i\hbar\gamma\begin{pmatrix} 1 & \cos\phi \\ \cos\phi & 1 \end{pmatrix}. \quad (1)$$

In (1), U is the direct coupling rate, $\hbar\gamma$ is the rate of radiative losses in the structure, and the off-diagonal term $\hbar\gamma\cos\phi$ represents the coupling between the two photon modes via the radiative continuum (far-field coupling), with the phase difference between two modes given by

$$\cos\phi = \frac{k_x^2 + k_y^2 - \frac{4\pi^2}{a^2}}{\sqrt{(k_x^2+k_y^2+\frac{4\pi^2}{a^2})^2 - 4k_x^2\frac{4\pi^2}{a^2}}}.$$

At small wavevectors ($k_{x,y} \ll 2\pi/a$) one gets

$$\cos\phi \approx -1 + k_y^2 a^2/2\pi^2 \to -1.$$

After diagonalizing the Hamiltonian (1), we obtain expressions for the new normal photonic modes in the etched waveguide:

$$E_{\rm C}^\pm = \hbar\omega_0 - i\hbar\gamma + \frac{\hbar c a}{4\pi n_{\rm g}}k_y^2 \pm \sqrt{\left(\frac{\hbar c k_x}{n_{\rm g}}\right)^2 + (U - i\hbar\gamma\cos\phi)^2}. \quad (2)$$

Expansion of (2) in the vicinity of $k_x, k_y = 0$ provides highly anisotropic effective masses of a photon in the x– and y–directions, respectively,

$$\frac{1}{m_{\rm C}^{x\pm}} = \pm{\rm Re}\left[\frac{c^2}{n_{\rm g}^2\sqrt{(U-i\hbar\gamma\cos\phi)^2}}\right], \quad \frac{1}{m_{\rm C}^{y\pm}} = \frac{ac}{2\pi\hbar n_{\rm g}}. \quad (3)$$

In the experimental fit of Ref. [2] $U < 0$ and

$$\frac{1}{m_{\rm C}^{x\pm}} = \pm\frac{c^2}{n_{\rm g}^2}\frac{|U|}{|U|^2 + |\hbar\gamma\cos\phi|^2}. \quad (4)$$

One can see that $m_{\rm C}^{x\pm}$ are dependent on momentum via $\cos\phi$, and for the "$-$" mode the effective photon mass in the x–direction is negative even without losses. In the following, we drop out the superscript "$-$" when referring to the effective mass of the "$-$" photon mode. Note that for relevant experimental parameters $|m_{\rm C}^x| \ll m_{\rm C}^y$.

For the "$-$" photon mode in (2) at small momenta

$$E_{\rm C}^-(p\sim 0) \approx \hbar\omega_0 - |U| + \frac{p_y^2}{2m_{\rm C}^y} + \frac{p_x^2}{2m_{\rm C}^x} - \frac{i\hbar\gamma}{U^2+\hbar\gamma^2}\frac{p_x^2c^2}{2n_{\rm g}^2},$$

which gives ${\rm Im}E_{\rm C}^- \to 0$ at $p_x \to 0$, i.e. this state is dark (which corresponds to the so-called bound state in the continuum, or BIC state).

Due to the symmetry reasons (the anti-node position of each photon mode corresponding to the node position of the other, see [2]), excitons in quantum wells cannot couple efficiently to both photonic modes at the same

* daniele.sanvitto@nanotec.cnr.it
* neenoune@gmail.com

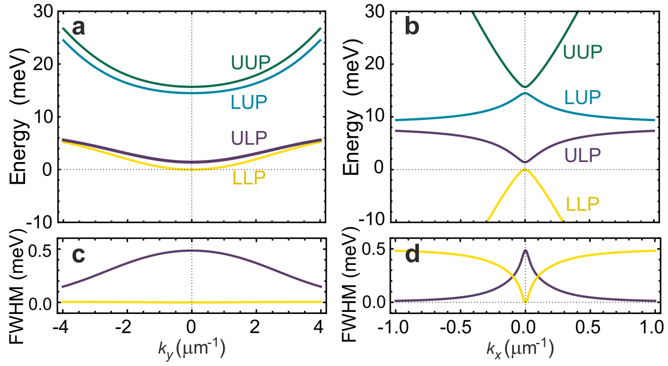

Supplementary Fig. 1: Exciton-polariton dispersions. (a,b) Four branches of the polariton dispersion (real parts) in a waveguide with grating: lower lower polariton (LLP, yellow) and upper lower polariton (ULP, purple) according to (7), lower upper polariton (LUP, blue) and upper upper polariton (UUP, green) versus k_y (a) and k_x (b), with the energy reference taken at the saddle point of the lowest mode. (c,d) Imaginary parts of the LLP and ULP energies (color code same as in top panels). Parameters are the same as used in the main text: $\Delta_0 = \hbar\omega_0 - E_{\rm g} = -0.6$ meV, $\hbar\Omega_{\rm R} = 15$ meV, $U = -1.3$ meV, $\hbar\gamma = 0.51$ meV, $n_{\rm g} = 4.3$, $m_{\rm X} = 0.22\ m_0$.

time. Thus we assume that the excitons with the standard isotropic parabolic dispersion

$$E_{\rm X}(k_x, k_y) = E_g + \hbar^2(k_x^2 + k_y^2)/2m_X - i\hbar\gamma_{\rm X} \qquad (5)$$

($E_{\rm g}$ gives the semiconductor energy gap, $m_{\rm X}$ is the exciton effective mass, and $\gamma_{\rm X} \ll \gamma$ is the non-radiative exciton decay rate) couple either to the "+" or the "–" photon modes in (2). In this case the 4×4 Hamiltonian of the system has the form:

$$\hat{H} = \begin{pmatrix} E_{\rm C}^{+} & \hbar\Omega_{\rm R}/2 & 0 & 0 \\ \hbar\Omega_{\rm R}/2 & E_{\rm X} & 0 & 0 \\ 0 & 0 & E_{\rm C}^{-} & \hbar\Omega_{\rm R}/2 \\ 0 & 0 & \hbar\Omega_{\rm R}/2 & E_{\rm X} \end{pmatrix}, \qquad (6)$$

where $\hbar\Omega_{\rm R}$ is the Rabi splitting energy. After the diagonalization of the Hamiltonian (6) we obtain two upper and two lower exciton-polariton modes corresponding to the two different ($\pm$) photon modes, which are shown in Fig. 1a,b as cuts along k_x and k_y.

As described in the main text, we assume that the upper polariton states (UUP and LUP in Supplementary Fig. 1) are not populated, while the dispersions of the two lower polariton branches are as follows:

$$E_{\pm}^{\rm LP} = \frac{E_{\rm C}^{\pm} + E_{\rm X}}{2} - \frac{1}{2}\sqrt{\left(E_{\rm C}^{\pm} - E_{\rm X}\right)^2 + (\hbar\Omega_{\rm R})^2}. \qquad (7)$$

The lower lower (LLP) and upper lower (LUP) polariton dispersions given by (7) are shown as yellow and purple lines in Supplementary Fig. 1, respectively, and their real parts are plotted in full three-dimensional view in Fig. 1b of the main text. At small momenta, the LLP dispersion is described by the asymptotic expression:

$$E_{-}^{\rm LP}(\mathbf{p} \sim 0) \approx \Delta_0 + \frac{p_x^2}{2m_{\rm LP}^x} + \frac{p_y^2}{2m_{\rm LP}^y} + i\frac{p_x^2}{2s^x} \qquad (8)$$

with

$$\frac{1}{m_{\rm LP}^{x(y)}} = \frac{C_0^2}{m_{\rm C}^{x(y)}} \quad \text{and} \quad \frac{1}{s^x} = -\frac{c^2}{n_{\rm g}^2}\frac{\hbar\gamma C_0^2}{U^2 + \hbar\gamma^2}.$$

Here $C_{\mathbf{p}}^2 = (1 - [\Delta_{\mathbf{p}} - U]/\sqrt{[\hbar\Omega_{\rm R}]^2 + [\Delta_{\mathbf{p}} - U]^2})/2$ is the photon Hopfield coefficient for the lower-polariton modes (generally complex, but the state $k = 0$ for the lower photon mode is dark, hence C_0^2 is real), and $\Delta_{\mathbf{p}} = E_{\rm C}^{-}(\mathbf{p}) - E_{\rm X}(\mathbf{p})$ is the photon-exciton detuning at a given momentum.

Non-radiative losses. The theoretical calculations presented in the main text are obtained in the neglection of the non-radiative exciton loss: $\gamma_{\rm X} \approx 0$. Curiously, when adding even a slight loss to the exciton dispersion in (5), the local minimum of the Bogoliubov dispersion at $k_x = 0$ which yields the regular linearization and the corresponding sound velocity, starts to shift and can eventually fully disappear. The characteristic behavior is shown in Supplementary Fig. 2: when $\gamma_{\rm X}$ is increased from 0, the saddle point shifts up. It needs to be pointed out that for physically relevant $\gamma_{\rm X}$ (here we took values from $10^{-3}\gamma$ to $10^{-2}\gamma$, see the colorful lines in Supplementary Fig. 2b), the local minimum of the dispersion in the region of small momenta still stays, but transforms into a "double-well" geometry (with the minima slightly shifted from 0 along k_x). The 3D view of the Bogoliubov dispersion around the saddle point for the case of finite non-radiative exciton loss rate is shown in Supplementary Fig. 2c and is to be compared to Fig. 4a of the main text. At further increase of $\gamma_{\rm X}$, the minimum could disappear completely tuning into a hill, however this case is not realized.

This curious behavior of the Bogoliubov dispersion at small momenta is clearly seen from Eq. (5) of the main text in the general case $\gamma_{\rm X} \neq 0$. In this case, the asymptotic formula (8) can be rewritten as

$$E_{-}^{\rm LP}(\mathbf{p} \sim 0) \approx \Delta_0 - i\hbar\Gamma + \frac{p_x^2}{2m_{\rm LP}^x} + \frac{p_y^2}{2m_{\rm LP}^y} + i\frac{p_x^2}{2s^x}$$

with

$$\hbar\Gamma = \frac{\hbar\gamma_{\rm X}}{2} + \frac{1}{2}{\rm Im}\sqrt{(\hbar\Omega_{\rm R})^2 + (\Delta - |U| + i\hbar\gamma_{\rm X})^2}.$$

Thus there are two essentially different cases corresponding to different momenta regions:

$$\begin{cases} \left|\left(\frac{p_x^2}{2m_{\rm LP}^x} + \frac{p_y^2}{2m_{\rm LP}^y} + i\frac{p_x^2}{2s^x}\right)\right| \ll |\hbar\Gamma|, \\ \left|\left(\frac{p_x^2}{2m_{\rm LP}^x} + \frac{p_y^2}{2m_{\rm LP}^y} + i\frac{p_x^2}{2s^x}\right)\right| \gg |\hbar\Gamma|. \end{cases}$$

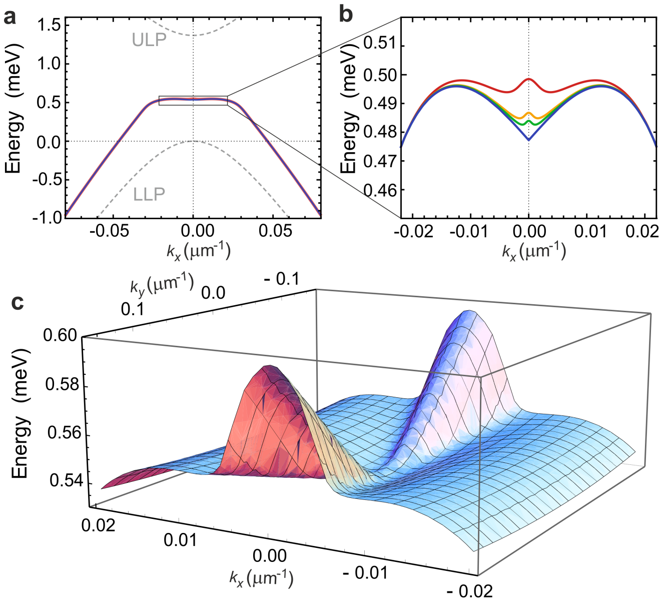

Supplementary Fig. 2: Influence of non-radiative exciton losses. (a,b) Real part of the Bogoliubov dispersion of excitations on top of the BIC-polariton condensate vs. k_x (the cut at $k_y = 0$) for different non-radiative exciton loss rates $\gamma_X = 0$ (blue), 0.001γ (green), 0.002γ (yellow), and 0.01γ (red). (c) Three-dimensional view of the dispersion of excitations zoomed in at the region of small momenta for $\gamma_X = 0.002\gamma$. In all panels, the condensate density $n_0 = 4 \times 10^{10}$ cm^{-2}, the dark reservoir density $\tilde{n} = 3 \times 10^{10}$ cm^{-2}, and exciton interaction constant $g = 2.5$ μeV μm^2.

In the first case, the approximate expression for the Bogoliubov spectrum reads:

$$E_{\mathbf{p}} \approx \sqrt{(2gn - i\hbar\Gamma)(-i\hbar\Gamma)} + \left(\frac{p_x^2}{2m_{\mathrm{LP}}^x} + \frac{p_y^2}{2m_{\mathrm{LP}}^y} + i\frac{p_x^2}{2s^x}\right)\frac{2gn - 2i\hbar\Gamma}{\sqrt{(2gn - i\hbar\Gamma)(-i\hbar\Gamma)}},$$

while for second case we obtain:

$$E_{\mathbf{p}} \approx \sqrt{(2gn - i\hbar\Gamma)\left(\frac{p_x^2}{2m_{\mathrm{LP}}^x} + \frac{p_y^2}{2m_{\mathrm{LP}}^y} + i\frac{p_x^2}{2s^x}\right)}.$$

From these equations one sees that the parabolic behavior at $k_x \sim 0$ changes to linear behavior with the increase of k_x. It is worth noting that the increase of non-radiative losses leads to vanishing of the linearization in the small-momenta region of the Bogoliubov dispersion in all directions. On the other hand, the difference of the spectrum in Supplementary Fig. 2 from the spectrum shown in the main text corresponds to an extremely narrow region around $k_x = 0$, which means it is unlikely to play a role in the reported experiment: to resolve such a narrow region in k, one would require a condensate spot of several hundreds of microns in diameter.

Temporal dynamics. Supplementary Figure 3 shows several snapshots extracted from the Supplementary Movie 1. Each panel represents the energy vs k_y emission taken at different times after the pulse arrival (the temporal delay is written on each panel of the figure). These snapshots display the cuts of the dispersion at $k_x \approx 0.02$ μm^{-1}, i.e. close to the saddle point of the LLP. The snapshots clearly show how the dispersion starts from a parabolic shape (blueshifted LLP) at earlier times, when the condensate is not yet formed and the ULP is populated (panel a), but becomes flat as polaritons start to accumulate in the BIC (panels b, c and d). As the condensation takes place, the emission from elementary excitations dispersion close to the condensate becomes dominant. As soon as the condensate starts fading the lower polariton branch recovers its parabolic shape (panels e and f), indicating that the flat region visible on panels b, c and d is related to the excitation spectrum of the condensate in the BIC, which shifts down in time (representing decreasing blueshift) as the particles are leaving the system.

Photoluminescence (k_x, k_y) scan below threshold. To better illustrate a drastic change that the energy-momentum dispersion undergoes when the condensate appears at the saddle point, here we show the experimental PL maps in (k_x, k_y) domain below threshold (see Supplementary Fig. 4) for three different energies: just below the saddle point of the LLP dispersion (a), at the saddle point (cutting at the BIC state, hence displaying a dark middle, panel b), and above the saddle point (c) where one can see a much higher populated ULP. Corresponding maps above threshold (at the level of the blueshifted BIC condensate and above) are shown in Fig. 3 of the main text. Supplementary Movies 2 and 3 show a sequence of such cuts for different energies above and below threshold, respectively.

Applicability. All theoretical calculations presented here are performed within the mean-field approximation that is applicable when $n'/n \ll 1$, where

$$n' = \sum_{\mathbf{p}\neq 0} |v_{\mathbf{p}}|^2 = \sum_{\neq 0} \frac{1}{2}\left|\sqrt{\frac{[\varepsilon_-(\mathbf{p}) + \mu_{\mathrm{LP}}]^2}{\varepsilon_-(\mathbf{p})[\varepsilon_-(\mathbf{p}) + 2\mu_{\mathrm{LP}}]}} - 1\right|$$

is the non-condensate lower polariton density and n is the total density. For the parameters used here (the considered values of density and interaction strength), the Bogoliubov approximation works well ($n'/n \sim 10^{-3}$).

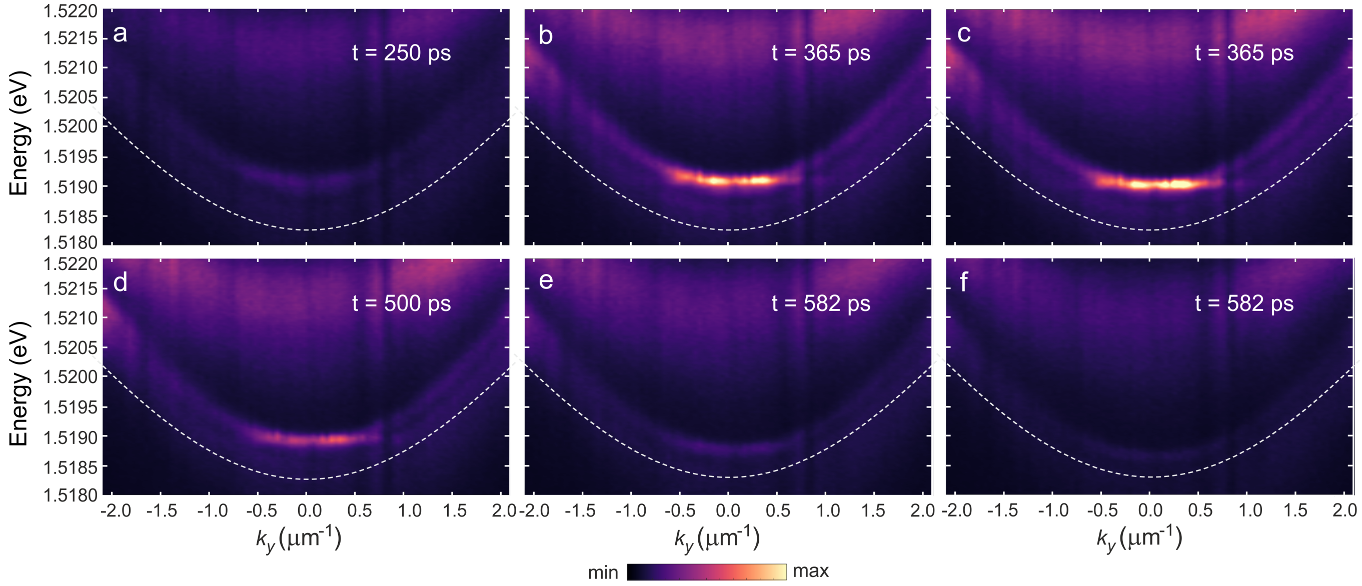

Supplementary Fig. 3: Time-resolved dynamics of the system energy dispersion. (a–f) Energy vs k_y emission close to the BIC state (cross-cuts of the dispersion at $k_x \approx 0.02\ \mu\text{m}^{-1}$), measured at different times after the arrival of the excitation pulse. Intensity color scale is in arbitrary units with the same normalisation for all panels, coinciding with that of Fig. 2d,e in the main text. The dashed lines show the non-blueshifted single-particle dispersion E_{-}^{LP} (7) (parameters as in Suppl. Fig. 1).

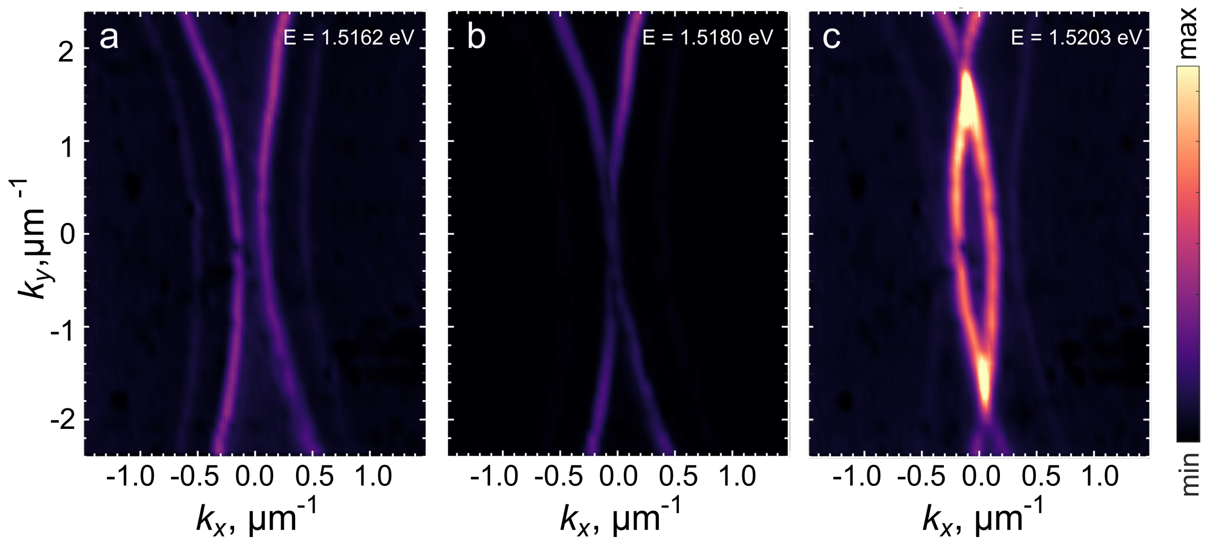

Supplementary Fig. 4: Momentum-space cuts below threshold. Experimental PL maps in the (k_x, k_y) domain taken at different energies below threshold, below the BIC energy (a), at the energy of the BIC (b) and above the BIC state (c). Intensity color scale is in arbitrary units with the same normalisation for all panels, coinciding with that of Fig. 4 in the main text. Compared to the PL maps shown in the main text, one can see a clear change of the dispersion around the saddle point, with the two-lobe pattern appearing only above threshold. For a more complete scan, see the Supplementary Movie 3.

SUPPLEMENTARY REFERENCES

[1] L. Lu, Q. Le-Van, L. Ferrier, E. Drouard, C. Seassal, and H. S. Nguyen, "Engineering a light–matter strong coupling regime in perovskite-based plasmonic metasurface: quasi-bound state in the continuum and exceptional points", Photon. Res. **8**(12), A91-A100 (2020).

[2] V. Ardizzone, F. Riminucci, S. Zanotti, A. Gianfrate, D. G. Suarez-Forero, F. Todisco, M. De Giorgi, D.Trypogeorgos, G. Gigli, H.S. Nguyen, K. Baldwin, L. Pfeiffer, D. Ballarini, D. Gerace, D. Sanvitto, "Polariton Bose-Einstein condensate from a Bound State in the Continuum", *Nature* **605**, 447-–452 (2022).
