# Supplementary material for: Collective excitations of a bound-in-the-continuum condensate
Source: Nat Commun. 2023 Jun 12;14:3464. doi: 10.1038/s41467-023-38939-y (PMC10261147; doi:10.1038/s41467-023-38939-y)
Supplement: Supplementary file 3 — Description of Additional Supplementary Files [file 41467_2023_38939_MOESM3_ESM.pdf]

File name: Supplementary Movie 1
Description: Example of temporal dynamics in the Energy vs. k_y space, reconstructed from the set of temporal traces. The time resolved measurements were obtained by scanning the far-field emission and acquiring for each k_y an Energy vs. time temporal trace with a streak camera.

File name: Supplementary Movie 2
Description: Sequence of cuts of the emission in the (k_x,k_y)–plane at different energies above threshold (like the images shown in Fig. 4). The data is obtained by scanning the far-field emission and acquiring a set of Energy vs. k_x dispersions, each one corresponding to a different k_y, and then merged and cut at a given energy in the plane k_x, k_y.

File name: Supplementary Movie 3
Description: Sequence of cuts of the emission in the (k_x,k_y)–plane at different energies below threshold (like the images shown in Supplementary Fig. 3). The data is obtained by scanning the far-field emission and acquiring a set of Energy vs. k_x dispersions, each one corresponding to a different k_y, and then merged and cut at a given energy in the plane k_x, k_y.
